# Pellagra and Uncinariasis in America

**Published:** 1910-06-11

**Authors:** 


### PELLAGRA AND UNCINARIASIS IN AMERICA.

Recent researches tend more and more to show that malaria has not in reality been so serious a factor in the south of the United States of America as was formerly supposed. Even some years ago it was strongly suspected that a considerable number of cases which had been diagnosed as malaria were in reality due to Eberth's bacillus, and this is now being proven through more systematic cytological examination of the blood for plasmodia, and its chemical examination by the Widal test, which has shown that typhoid fever is pandemic throughout the United States.

### The "Diploma Mill" Quack.

After the Civil War in 1864 the South was much impoverished, and the education of its people suffered greatly. Its citizens thus fell an easy prey to the "diploma mill" doctor, who at one time was a scandal to the medical profession. The most natural course was the easy one of encouraging the Southerners' belief in malaria as an explanation of any indisposition. Cases of mild gastric, hepatic, or metabolic disturbance were thus diagnosed without even a physical examination of the patient. The Southerner is very subject to "bilious attacks" of this kind, on account of his partiality to a diet anything but suitable to a climate which is not only moist and warm, but exceedingly changeable. The countryman's staple food is insufficiently cooked bacon and imperfectly baked soda scones of finely bolted flour, eaten hot. Fruit and greens are only taken occasionally, the sweet potato being the vegetable principally eaten.

This class of case and the abortive and anomalous typhoid made up a large proportion of the malaria diagnosed; and recently the total even of the chronic cases has been lessened by the identification of two affections which are now found to prevail quite extensively in the South and Middle West.

These are pellagra and uncinariasis. The former, a disease of maize-eating people, has been long known in Italy, where it is believed to be due to a mold growing upon damp maize.

It was not, however, even suspected to be in the United States until last year, although Indian corn is the staple cereal of the negro population and many whites, both in the South and West. It was first found at a State Asylum in Carolina, for it often produces mental unsoundness by its toxic action on the cerebral neurones. It has now been found to be quite extensive throughout the corn belt, even so far north as Chicago, where at the city poorhouse alone 26 cases were treated, of whom 14 died. So seriously is the disease regarded that Surgeon-General Wyman, of the Marine Hospital and Public Health Service, has called a conference this year, to which have been invited leading southern physicians and representatives of the Services in order to co-ordinate the data they have collected and to devise plans to study the ætiology and prevention of a disease the spread of which would seriously interfere with the renewal of prosperity in the Southern States.

In connection with the Commission, much indignation has been expressed that Major Ashworth, who discovered the uncinaria in 1899, has not been appointed to the Commission. He is, however, on duty in Porto Rico, where he conducted his first investigations after observing that three-fourths of the cases of anæmia in the hospitals there did not improve with the rest and good nutrition prescribed and given them. He suspected the parasite, and found the ova in the stools.

It is believed that in reality the disease has existed for many years, but that readiness to diagnose malaria, and ignorance of psychiatrical diagnosis, and especially the placing apart of mental alienation study from that of general pathology and medicine, hindered its detection.

Both the preceding disease and the presence of the hook-worm parasite are at present very much in the public eye in America, the latter largely on account of a recent gift of Mr. Rockefeller of over two hundred thousand dollars to endow an institute for its study. The main credit for the attention it has excited for some five years is due to Stiles, the zoologist of the Marine Hospital Service.

Previous to this, it had excited public attention on account of its bearing on the condition of the cotton factories of the Carolinas and Georgia. It is found to be appallingly rife there, and to be largely responsible for the indolence of the poor white of the South (hence the name "lazy-man's disease"), neither the climate nor weather being the real cause.

### Signs of Improvement.

The conditions are particularly unfavourable because of the stubborn pride of the southern white, a legacy from Cavalier days, in consequence of which he cannot be forced to take measures which his shiftlessness prevents him undertaking voluntarily. It is hoped that the industrial development in the Carolina hills may increase enlightenment to such a degree that community action may become less difficult and more intelligent than would have been possible to such people as the wealthy farmer who once declared to the writer, *à propos* of the propagation of malaria by the mosquito, "If you talk to the Day of Judgment, I could never believe that. It is contrary to nature."

The work of the Marine Hospital Service in demonstrating by lantern-slides and specimens the story of the disease bids fair to make an end of this unprogressive attitude, and legislative measures are already being advocated against the scourge. So much is this so that Dr. Stiles advocates the continuance of the employment of the children in the mills recently forbidden by a child-labour law. He believes that they are healthier under the care of a considerate manufacturer than in the inexpressibly insanitary homes of their parents. This surprising proposal is an index to the foreign reader of the extraordinary gravity of the situation in the eyes of those by whom it is being studied most. The results formulated by the organisations which are undertaking the fight against the two diseases may in the future have considerable importance to the British possessions in South Africa, Asia, and Australia.

